# Pdx1 regulates pancreas tubulogenesis and E-cadherin expression

**DOI:** 10.1242/dev.135806

**Published:** 2016-03-15

**Authors:** Leilani Marty-Santos, Ondine Cleaver

There was an error published in Development **143**, 101-112.

The authors omitted to mention the mouse line pdx^lacZko+/−^, which was generated and provided by Dr Christopher Wright (Offield et al., 1996). This mouse mutant constituted the basis of their study.

The authors apologise to readers for this mistake.

